# [*N*,*N*′-Bis(3-meth­oxy-2-oxidobenzyl­idene)cyclo­hexane-1,2-diaminium-κ^4^ *O*,*O*′,*O*′′,*O*′′′]tris­(nitrato-κ^2^ *O*,*O*′)europium(III) methanol monosolvate

**DOI:** 10.1107/S1600536810046076

**Published:** 2010-11-24

**Authors:** Peng-Fei Yan, Yan Bao, Guang-Ming Li, Jing-Ya Li, Peng Chen

**Affiliations:** a School of Chemistry and Materials Science, Heilongjiang University, Harbin 150080, People’s Republic of China

## Abstract

In the title mononuclear salen-type complex, [Eu(NO_3_)_3_(C_22_H_26_N_2_O_4_)]·CH_3_OH, the Eu^III^ ion is ten-coordinated by three bidentate nitrate counter-ions and one organic salen-type ligand, which acts in a bis-bidentate chelating mode through its phenolate and meth­oxy O atoms. The protonated imine groups are involved in intra­molecular N—H⋯O hydrogen bonds to the phenolate O atomss, emphasizing the zwitterionic nature of the ligand. An O—H⋯O hydrogen bond links the complex and solvent mol­ecules.

## Related literature

For the synthesis of the salen-type ligand, see: Mohamed *et al.* (2003 ▶); Aslantaş *et al.* (2007) ▶. For the synthesis of lanthanide complexes with a similar ligand, see: Yang *et al.* (2006 ▶, 2008 ▶).
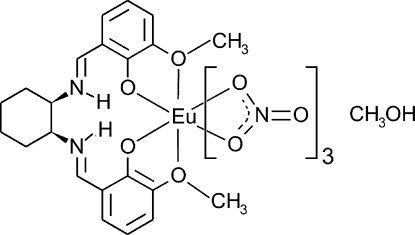


## Experimental

### 

#### Crystal data

[Eu(NO_3_)_3_(C_22_H_26_N_2_O_4_)]·CH_4_O
*M* *_r_* = 752.48
Triclinic, 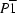

*a* = 9.7718 (4) Å
*b* = 12.8560 (6) Å
*c* = 13.0567 (6) Å
α = 78.798 (1)°
β = 68.492 (1)°
γ = 81.671 (1)°
*V* = 1492.09 (12) Å^3^
*Z* = 2
Mo *K*α radiation
μ = 2.18 mm^−1^
*T* = 291 K
0.40 × 0.22 × 0.20 mm

#### Data collection

Rigaku R-AXIS RAPID diffractometer
Absorption correction: multi-scan (*ABSCOR*; Higashi, 1995 ▶) *T*_min_ = 0.476, *T*_max_ = 0.670
8377 measured reflections
5185 independent reflections
4683 reflections with *I* > 2σ(*I*)
*R*_int_ = 0.011

#### Refinement

*R*[*F* ^2^ > 2σ(*F* ^2^)] = 0.025
*wR*(*F* ^2^) = 0.068
*S* = 1.02
5185 reflections
390 parameters
H-atom parameters constrained
Δρ_max_ = 0.76 e Å^−3^
Δρ_min_ = −0.72 e Å^−3^

### 

Data collection: *RAPID-AUTO* (Rigaku, 1998 ▶); cell refinement: *RAPID-AUTO*; data reduction: *RAPID-AUTO*; program(s) used to solve structure: *SHELXS97* (Sheldrick, 2008 ▶); program(s) used to refine structure: *SHELXL97* (Sheldrick, 2008 ▶); molecular graphics: *SHELXTL* (Sheldrick, 2008 ▶); software used to prepare material for publication: *SHELXL97*.

## Supplementary Material

Crystal structure: contains datablocks global, I. DOI: 10.1107/S1600536810046076/gk2295sup1.cif

Structure factors: contains datablocks I. DOI: 10.1107/S1600536810046076/gk2295Isup2.hkl

Additional supplementary materials:  crystallographic information; 3D view; checkCIF report

## Figures and Tables

**Table 1:** Hydrogen-bond geometry (Å, °)

*D*—H⋯*A*	*D*—H	H⋯*A*	*D*⋯*A*	*D*—H⋯*A*
N1—H1*N*⋯O1	0.86	1.88	2.575 (3)	137
N2—H2*N*⋯O3	0.86	1.88	2.593 (3)	139
O1*M*—H1*O*⋯O13	0.85	2.18	2.993 (6)	160

**Fig. 1.**
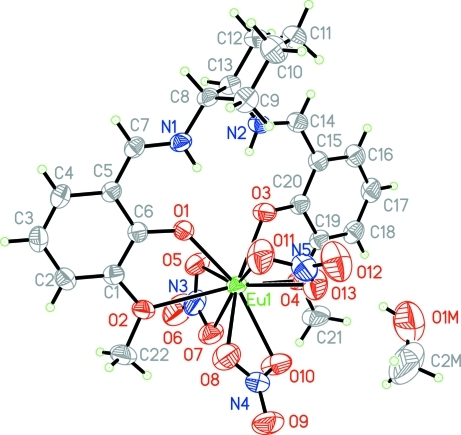
The molecular structure of the title compound, showing 40% probability displacement ellipsoids.

## supplementary crystallographic information

### Comment

We present here the crystal structure of the title compound. As shown in Fig. 1, the Eu(III) ion is ten-coordinated by three bidentate nitrate counterions and one ligand that utilizes two hydroxyl oxygen atoms and two methoxy oxygen atoms, while the nitrogen atoms remain protonated (Yang *et al.*, 2006, 2008). The Eu—O bond lengths are in the range of 2.493 (3)–2.604 (3) Å. See Yang *et al.* 2006 and Yang *et al.* (2008) for the synthesis of lanthanide complex with *N*,*N*'-bis(5-bromo-3-methoxysalicylidene)phenylene-1,2-cyclohexanediamine ligand.

### Experimental

To a CH_2_Cl_2_ solution (5 ml) of H_2_L (0.0368 g, 0.1 mmol) under stirring was slowly added a MeCN (5 ml)/MeOH (5 ml) solution of Eu(NO_3_)_3_˙6H_2_O (0.0446 g, 0.1 mmol) at room temperature. The diethyl ether was allowed to diffuse slowly into the filtrate at room temperature. The light yellow crystals were obtained within one week. [(Eu(H_2_L)(NO_3_)_3_]ĊH_3_OH Elemental Anal. Calc. for C_23_H_30_N_5_O_14_Eu: C, 36.71; H, 4.02; N, 9.31 %, Found: C, 36.78; H, 4.11; N, 9.32 wt%.

### Refinement

All H atoms were placed in calculated positions and treated as riding on their parent atoms, with C—H = 0.93-0.97 Å , N-H = 0.86 Å, O-H = 0.85 and *U*_iso_(H) = 1.2 *U*_eq_(C, N) or *U*_iso_(H) = 1.5 *U*_eq_(O, C_methyl_).

### Crystal data

**Table:**

[Eu(NO_3_)_3_(C_22_H_26_N_2_O_4_)]·CH_4_O	*Z* = 2
*M**_r_* = 752.48	*F*(000) = 756
Triclinic, *P*1	*D*_x_ = 1.675 Mg m^−^^3^
Hall symbol: -P 1	Mo *K*α radiation, λ = 0.71073 Å
*a* = 9.7718 (4) Å	Cell parameters from 21234 reflections
*b* = 12.8560 (6) Å	θ = 2.5–28.3°
*c* = 13.0567 (6) Å	µ = 2.18 mm^−^^1^
α = 78.798 (1)°	*T* = 291 K
β = 68.492 (1)°	Block, colorless
γ = 81.671 (1)°	0.40 × 0.22 × 0.20 mm
*V* = 1492.09 (12) Å^3^	

### Data collection

**Table:**

Rigaku R-AXIS RAPID diffractometer	5185 independent reflections
Radiation source: fine-focus sealed tube	4683 reflections with *I* > 2σ(*I*)
graphite	*R*_int_ = 0.011
ω scans	θ_max_ = 25.0°, θ_min_ = 2.7°
Absorption correction: multi-scan (*ABSCOR*; Higashi, 1995)	*h* = −10→11
*T*_min_ = 0.476, *T*_max_ = 0.670	*k* = −14→15
8377 measured reflections	*l* = 0→15

### Refinement

**Table:**

Refinement on *F*^2^	Primary atom site location: structure-invariant direct methods
Least-squares matrix: full	Secondary atom site location: difference Fourier map
*R*[*F*^2^ > 2σ(*F*^2^)] = 0.025	Hydrogen site location: inferred from neighbouring sites
*wR*(*F*^2^) = 0.068	H-atom parameters constrained
*S* = 1.02	*w* = 1/[σ^2^(*F*_o_^2^) + (0.0397*P*)^2^ + 0.6408*P*] where *P* = (*F*_o_^2^ + 2*F*_c_^2^)/3
5185 reflections	(Δ/σ)_max_ = 0.002
390 parameters	Δρ_max_ = 0.76 e Å^−^^3^
0 restraints	Δρ_min_ = −0.72 e Å^−^^3^

### Special details

**Table:**

Geometry. All esds (except the esd in the dihedral angle between two l.s. planes) are estimated using the full covariance matrix. The cell esds are taken into account individually in the estimation of esds in distances, angles and torsion angles; correlations between esds in cell parameters are only used when they are defined by crystal symmetry. An approximate (isotropic) treatment of cell esds is used for estimating esds involving l.s. planes.
Refinement. Refinement of F^2^ against ALL reflections. The weighted R-factor wR and goodness of fit S are based on F^2^, conventional R-factors R are based on F, with F set to zero for negative F^2^. The threshold expression of F^2^ > 2sigma(F^2^) is used only for calculating R-factors(gt) etc. and is not relevant to the choice of reflections for refinement. R-factors based on F^2^ are statistically about twice as large as those based on F, and R- factors based on ALL data will be even larger.

### Fractional atomic coordinates and isotropic or equivalent isotropic displacement parameters (Å^2^)

**Table:**

	*x*	*y*	*z*	*U*_iso_*/*U*_eq_	
Eu1	0.334321 (18)	0.769873 (11)	0.296926 (12)	0.04613 (7)	
O1	0.2712 (3)	0.64308 (17)	0.45525 (18)	0.0583 (6)	
O2	0.2513 (3)	0.83055 (17)	0.5057 (2)	0.0604 (6)	
O3	0.4470 (3)	0.60529 (17)	0.24850 (18)	0.0577 (6)	
O4	0.5486 (3)	0.77389 (17)	0.10733 (18)	0.0545 (6)	
O5	0.5635 (3)	0.7515 (2)	0.3459 (2)	0.0717 (7)	
O6	0.6825 (5)	0.8845 (3)	0.3390 (4)	0.1204 (15)	
O7	0.4880 (3)	0.9107 (2)	0.2920 (2)	0.0691 (7)	
O8	0.1193 (4)	0.9066 (3)	0.3312 (2)	0.0882 (10)	
O9	0.0929 (4)	1.0474 (3)	0.2154 (3)	0.0978 (11)	
O10	0.2917 (3)	0.9440 (2)	0.1769 (3)	0.0772 (8)	
O11	0.1063 (4)	0.6753 (3)	0.3153 (3)	0.0897 (10)	
O12	0.0405 (4)	0.6683 (3)	0.1759 (3)	0.1056 (12)	
O13	0.2334 (3)	0.7438 (2)	0.1540 (2)	0.0750 (8)	
N1	0.2408 (3)	0.4421 (2)	0.5122 (2)	0.0457 (6)	
H1N	0.2568	0.4975	0.4618	0.055*	
N2	0.4602 (3)	0.4004 (2)	0.3056 (2)	0.0462 (6)	
H2N	0.4272	0.4635	0.3204	0.055*	
N3	0.5814 (4)	0.8503 (3)	0.3258 (3)	0.0734 (10)	
N4	0.1652 (4)	0.9683 (2)	0.2404 (3)	0.0624 (8)	
N5	0.1231 (4)	0.6948 (3)	0.2153 (3)	0.0671 (9)	
C1	0.2144 (3)	0.7496 (2)	0.5949 (3)	0.0440 (7)	
C2	0.1654 (4)	0.7602 (3)	0.7053 (3)	0.0505 (8)	
H2	0.1561	0.8268	0.7261	0.061*	
C3	0.1291 (4)	0.6694 (3)	0.7874 (3)	0.0568 (9)	
H3	0.0949	0.6766	0.8624	0.068*	
C4	0.1438 (4)	0.5718 (3)	0.7579 (3)	0.0505 (8)	
H4	0.1201	0.5127	0.8129	0.061*	
C5	0.1950 (3)	0.5590 (2)	0.6442 (2)	0.0410 (7)	
C6	0.2288 (3)	0.6498 (2)	0.5614 (2)	0.0424 (7)	
C7	0.2065 (3)	0.4573 (2)	0.6140 (2)	0.0433 (7)	
H7	0.1886	0.3986	0.6700	0.052*	
C8	0.2548 (3)	0.3397 (2)	0.4746 (3)	0.0426 (7)	
H8	0.2302	0.2848	0.5407	0.051*	
C9	0.1478 (4)	0.3382 (3)	0.4160 (3)	0.0567 (9)	
H9A	0.1680	0.3928	0.3509	0.068*	
H9B	0.0478	0.3532	0.4655	0.068*	
C10	0.1627 (4)	0.2281 (3)	0.3806 (4)	0.0680 (10)	
H10A	0.1324	0.1748	0.4465	0.082*	
H10B	0.0977	0.2291	0.3392	0.082*	
C11	0.3190 (4)	0.1984 (3)	0.3094 (3)	0.0595 (9)	
H11A	0.3442	0.2457	0.2388	0.071*	
H11B	0.3260	0.1265	0.2943	0.071*	
C12	0.4284 (4)	0.2050 (3)	0.3651 (3)	0.0527 (8)	
H12A	0.5277	0.1914	0.3138	0.063*	
H12B	0.4123	0.1502	0.4299	0.063*	
C13	0.4150 (3)	0.3129 (2)	0.4009 (3)	0.0447 (7)	
H13	0.4784	0.3087	0.4451	0.054*	
C14	0.5425 (3)	0.3944 (3)	0.2032 (3)	0.0473 (7)	
H14	0.5730	0.3275	0.1817	0.057*	
C15	0.5883 (3)	0.4856 (3)	0.1224 (3)	0.0463 (7)	
C16	0.6903 (4)	0.4733 (3)	0.0152 (3)	0.0574 (9)	
H16	0.7188	0.4057	−0.0046	0.069*	
C17	0.7467 (4)	0.5591 (3)	−0.0591 (3)	0.0615 (9)	
H17	0.8148	0.5496	−0.1289	0.074*	
C18	0.7038 (4)	0.6623 (3)	−0.0320 (3)	0.0568 (9)	
H18	0.7449	0.7205	−0.0833	0.068*	
C19	0.6014 (4)	0.6770 (3)	0.0700 (3)	0.0471 (7)	
C20	0.5417 (4)	0.5890 (3)	0.1506 (3)	0.0460 (7)	
C21	0.6066 (5)	0.8667 (3)	0.0304 (4)	0.0790 (13)	
H21A	0.7119	0.8618	0.0104	0.119*	
H21B	0.5643	0.9291	0.0646	0.119*	
H21C	0.5823	0.8713	−0.0354	0.119*	
C22	0.2283 (5)	0.9369 (3)	0.5309 (4)	0.0775 (12)	
H22A	0.1264	0.9517	0.5740	0.116*	
H22B	0.2548	0.9864	0.4628	0.116*	
H22C	0.2886	0.9439	0.5727	0.116*	
O1M	0.3110 (9)	0.8258 (6)	−0.0896 (5)	0.190 (3)	
H1O	0.3098	0.7939	−0.0258	0.285*	
C2M	0.204 (2)	0.9063 (7)	−0.0741 (11)	0.303 (11)	
H2MA	0.1879	0.9313	−0.1431	0.363*	
H2MB	0.2391	0.9638	−0.0555	0.363*	
H2MC	0.1121	0.8885	−0.0171	0.454*	

### Atomic displacement parameters (Å^2^)

**Table:**

	*U*^11^	*U*^22^	*U*^33^	*U*^12^	*U*^13^	*U*^23^
Eu1	0.05387 (12)	0.03330 (10)	0.03868 (10)	−0.00222 (7)	−0.00491 (7)	0.00021 (6)
O1	0.0848 (18)	0.0388 (12)	0.0358 (12)	−0.0081 (11)	−0.0029 (12)	−0.0032 (9)
O2	0.0812 (18)	0.0363 (12)	0.0516 (14)	−0.0032 (11)	−0.0097 (13)	−0.0063 (10)
O3	0.0671 (15)	0.0384 (12)	0.0412 (12)	−0.0019 (11)	0.0092 (11)	−0.0029 (10)
O4	0.0574 (14)	0.0414 (12)	0.0456 (13)	−0.0078 (10)	−0.0013 (11)	0.0068 (10)
O5	0.0819 (19)	0.0623 (17)	0.0714 (18)	−0.0032 (14)	−0.0359 (16)	0.0056 (14)
O6	0.136 (3)	0.121 (3)	0.136 (3)	−0.057 (3)	−0.091 (3)	0.027 (3)
O7	0.0823 (19)	0.0531 (15)	0.0707 (17)	−0.0138 (14)	−0.0300 (16)	0.0060 (13)
O8	0.085 (2)	0.090 (2)	0.0584 (17)	0.0272 (17)	−0.0053 (16)	−0.0025 (16)
O9	0.105 (3)	0.076 (2)	0.097 (2)	0.0413 (19)	−0.037 (2)	−0.0086 (17)
O10	0.0685 (18)	0.0566 (16)	0.081 (2)	0.0073 (14)	−0.0117 (16)	0.0106 (14)
O11	0.089 (2)	0.112 (3)	0.0550 (18)	−0.0422 (19)	−0.0033 (16)	0.0015 (17)
O12	0.092 (2)	0.138 (3)	0.095 (3)	−0.055 (2)	−0.018 (2)	−0.033 (2)
O13	0.0728 (18)	0.092 (2)	0.0536 (16)	−0.0344 (16)	−0.0083 (14)	−0.0040 (14)
N1	0.0498 (15)	0.0358 (13)	0.0431 (15)	−0.0063 (11)	−0.0094 (12)	0.0018 (11)
N2	0.0430 (15)	0.0362 (13)	0.0502 (16)	−0.0009 (11)	−0.0060 (12)	−0.0074 (11)
N3	0.086 (3)	0.077 (2)	0.059 (2)	−0.023 (2)	−0.032 (2)	0.0085 (18)
N4	0.075 (2)	0.0479 (17)	0.063 (2)	0.0108 (16)	−0.0255 (18)	−0.0141 (15)
N5	0.067 (2)	0.066 (2)	0.061 (2)	−0.0219 (17)	−0.0061 (18)	−0.0137 (16)
C1	0.0397 (17)	0.0422 (17)	0.0463 (18)	−0.0026 (13)	−0.0117 (14)	−0.0052 (14)
C2	0.0482 (19)	0.0532 (19)	0.051 (2)	0.0022 (15)	−0.0163 (16)	−0.0171 (16)
C3	0.059 (2)	0.070 (2)	0.0387 (18)	−0.0025 (18)	−0.0134 (16)	−0.0120 (17)
C4	0.0498 (19)	0.058 (2)	0.0388 (17)	−0.0090 (15)	−0.0123 (15)	0.0011 (15)
C5	0.0355 (16)	0.0433 (16)	0.0389 (16)	−0.0029 (13)	−0.0090 (13)	−0.0023 (13)
C6	0.0381 (16)	0.0440 (17)	0.0391 (16)	−0.0029 (13)	−0.0071 (13)	−0.0053 (13)
C7	0.0385 (16)	0.0468 (18)	0.0374 (16)	−0.0062 (13)	−0.0082 (13)	0.0022 (13)
C8	0.0455 (17)	0.0353 (15)	0.0413 (17)	−0.0070 (13)	−0.0111 (14)	0.0023 (13)
C9	0.0453 (19)	0.063 (2)	0.058 (2)	−0.0027 (16)	−0.0167 (17)	−0.0048 (17)
C10	0.061 (2)	0.073 (3)	0.081 (3)	−0.019 (2)	−0.030 (2)	−0.016 (2)
C11	0.068 (2)	0.051 (2)	0.065 (2)	−0.0102 (17)	−0.0254 (19)	−0.0117 (17)
C12	0.055 (2)	0.0382 (17)	0.060 (2)	−0.0003 (15)	−0.0181 (17)	−0.0044 (15)
C13	0.0435 (17)	0.0397 (16)	0.0484 (18)	−0.0045 (13)	−0.0156 (14)	−0.0006 (13)
C14	0.0428 (18)	0.0460 (18)	0.0505 (19)	−0.0011 (14)	−0.0125 (15)	−0.0107 (15)
C15	0.0415 (17)	0.0490 (18)	0.0417 (17)	0.0014 (14)	−0.0074 (14)	−0.0094 (14)
C16	0.054 (2)	0.064 (2)	0.0452 (19)	0.0036 (17)	−0.0063 (16)	−0.0146 (17)
C17	0.054 (2)	0.078 (3)	0.0376 (18)	0.0034 (19)	−0.0008 (16)	−0.0121 (18)
C18	0.049 (2)	0.068 (2)	0.0409 (18)	−0.0081 (17)	−0.0058 (15)	0.0029 (16)
C19	0.0440 (18)	0.0487 (18)	0.0402 (17)	−0.0019 (14)	−0.0079 (14)	−0.0016 (14)
C20	0.0439 (18)	0.0467 (18)	0.0389 (17)	−0.0031 (14)	−0.0070 (14)	−0.0020 (14)
C21	0.088 (3)	0.049 (2)	0.066 (3)	−0.015 (2)	0.004 (2)	0.0148 (19)
C22	0.106 (3)	0.0391 (19)	0.074 (3)	−0.003 (2)	−0.015 (2)	−0.0109 (18)
O1M	0.290 (9)	0.176 (6)	0.106 (4)	−0.019 (6)	−0.063 (5)	−0.038 (4)
C2M	0.67 (3)	0.119 (7)	0.301 (15)	−0.125 (12)	−0.39 (2)	0.051 (8)

### Geometric parameters (Å, °)

**Table:**

Eu1—O1	2.315 (2)	C5—C7	1.415 (4)
Eu1—O3	2.329 (2)	C5—C6	1.415 (4)
Eu1—O7	2.492 (3)	C7—H7	0.9300
Eu1—O8	2.500 (3)	C8—C9	1.509 (5)
Eu1—O13	2.501 (3)	C8—C13	1.537 (4)
Eu1—O5	2.513 (3)	C8—H8	0.9800
Eu1—O10	2.547 (3)	C9—C10	1.544 (5)
Eu1—O4	2.588 (2)	C9—H9A	0.9700
Eu1—O11	2.603 (3)	C9—H9B	0.9700
Eu1—O2	2.778 (2)	C10—C11	1.506 (5)
Eu1—N3	2.930 (4)	C10—H10A	0.9700
Eu1—N4	2.954 (3)	C10—H10B	0.9700
O1—C6	1.310 (4)	C11—C12	1.516 (5)
O2—C1	1.378 (4)	C11—H11A	0.9700
O2—C22	1.436 (4)	C11—H11B	0.9700
O3—C20	1.309 (4)	C12—C13	1.521 (4)
O4—C19	1.386 (4)	C12—H12A	0.9700
O4—C21	1.438 (4)	C12—H12B	0.9700
O5—N3	1.271 (4)	C13—H13	0.9800
O6—N3	1.216 (5)	C14—C15	1.419 (5)
O7—N3	1.260 (4)	C14—H14	0.9300
O8—N4	1.259 (4)	C15—C16	1.413 (5)
O9—N4	1.214 (4)	C15—C20	1.416 (4)
O10—N4	1.245 (4)	C16—C17	1.352 (5)
O11—N5	1.233 (4)	C16—H16	0.9300
O12—N5	1.219 (5)	C17—C18	1.405 (5)
O13—N5	1.252 (4)	C17—H17	0.9300
N1—C7	1.293 (4)	C18—C19	1.370 (5)
N1—C8	1.464 (4)	C18—H18	0.9300
N1—H1N	0.8600	C19—C20	1.416 (4)
N2—C14	1.293 (4)	C21—H21A	0.9600
N2—C13	1.485 (4)	C21—H21B	0.9600
N2—H2N	0.8600	C21—H21C	0.9600
C1—C2	1.370 (4)	C22—H22A	0.9600
C1—C6	1.406 (4)	C22—H22B	0.9600
C2—C3	1.411 (5)	C22—H22C	0.9600
C2—H2	0.9300	O1M—C2M	1.346 (15)
C3—C4	1.355 (5)	O1M—H1O	0.8506
C3—H3	0.9300	C2M—H2MA	0.9600
C4—C5	1.418 (4)	C2M—H2MB	0.9599
C4—H4	0.9300	C2M—H2MC	0.9600
			
O1—Eu1—O3	71.40 (8)	O11—N5—Eu1	60.2 (2)
O1—Eu1—O7	117.10 (9)	O13—N5—Eu1	55.6 (2)
O3—Eu1—O7	119.72 (9)	C2—C1—O2	126.3 (3)
O1—Eu1—O8	104.34 (9)	C2—C1—C6	121.3 (3)
O3—Eu1—O8	151.65 (12)	O2—C1—C6	112.3 (3)
O7—Eu1—O8	87.60 (11)	C1—C2—C3	119.6 (3)
O1—Eu1—O13	113.51 (9)	C1—C2—H2	120.2
O3—Eu1—O13	76.75 (10)	C3—C2—H2	120.2
O7—Eu1—O13	129.39 (9)	C4—C3—C2	120.6 (3)
O8—Eu1—O13	79.81 (11)	C4—C3—H3	119.7
O1—Eu1—O5	79.38 (9)	C2—C3—H3	119.7
O3—Eu1—O5	76.72 (9)	C3—C4—C5	120.7 (3)
O7—Eu1—O5	50.82 (9)	C3—C4—H4	119.6
O8—Eu1—O5	130.97 (11)	C5—C4—H4	119.6
O13—Eu1—O5	144.46 (10)	C7—C5—C6	120.5 (3)
O1—Eu1—O10	154.01 (9)	C7—C5—C4	120.4 (3)
O3—Eu1—O10	130.94 (9)	C6—C5—C4	119.0 (3)
O7—Eu1—O10	67.06 (10)	O1—C6—C1	119.7 (3)
O8—Eu1—O10	49.69 (10)	O1—C6—C5	121.6 (3)
O13—Eu1—O10	67.59 (10)	C1—C6—C5	118.7 (3)
O5—Eu1—O10	115.53 (9)	N1—C7—C5	123.1 (3)
O1—Eu1—O4	132.42 (8)	N1—C7—H7	118.5
O3—Eu1—O4	63.86 (7)	C5—C7—H7	118.5
O7—Eu1—O4	74.94 (9)	N1—C8—C9	111.3 (3)
O8—Eu1—O4	122.65 (8)	N1—C8—C13	110.4 (2)
O13—Eu1—O4	71.69 (8)	C9—C8—C13	111.8 (3)
O5—Eu1—O4	75.58 (9)	N1—C8—H8	107.7
O10—Eu1—O4	73.42 (8)	C9—C8—H8	107.7
O1—Eu1—O11	68.80 (10)	C13—C8—H8	107.7
O3—Eu1—O11	79.36 (11)	C8—C9—C10	109.9 (3)
O7—Eu1—O11	160.81 (11)	C8—C9—H9A	109.7
O8—Eu1—O11	73.22 (12)	C10—C9—H9A	109.7
O13—Eu1—O11	48.66 (9)	C8—C9—H9B	109.7
O5—Eu1—O11	144.90 (10)	C10—C9—H9B	109.7
O10—Eu1—O11	99.54 (11)	H9A—C9—H9B	108.2
O4—Eu1—O11	115.63 (9)	C11—C10—C9	111.5 (3)
O1—Eu1—O2	60.11 (7)	C11—C10—H10A	109.3
O3—Eu1—O2	126.21 (8)	C9—C10—H10A	109.3
O7—Eu1—O2	69.33 (8)	C11—C10—H10B	109.3
O8—Eu1—O2	67.85 (9)	C9—C10—H10B	109.3
O13—Eu1—O2	142.49 (9)	H10A—C10—H10B	108.0
O5—Eu1—O2	73.00 (9)	C10—C11—C12	112.1 (3)
O10—Eu1—O2	102.24 (9)	C10—C11—H11A	109.2
O4—Eu1—O2	142.38 (8)	C12—C11—H11A	109.2
O11—Eu1—O2	101.98 (9)	C10—C11—H11B	109.2
O1—Eu1—N3	98.64 (9)	C12—C11—H11B	109.2
O3—Eu1—N3	98.70 (11)	H11A—C11—H11B	107.9
O7—Eu1—N3	25.25 (9)	C11—C12—C13	112.5 (3)
O8—Eu1—N3	109.63 (12)	C11—C12—H12A	109.1
O13—Eu1—N3	143.40 (9)	C13—C12—H12A	109.1
O5—Eu1—N3	25.57 (9)	C11—C12—H12B	109.1
O10—Eu1—N3	91.20 (10)	C13—C12—H12B	109.1
O4—Eu1—N3	73.81 (9)	H12A—C12—H12B	107.8
O11—Eu1—N3	167.32 (10)	N2—C13—C12	113.2 (3)
O2—Eu1—N3	68.89 (9)	N2—C13—C8	108.9 (2)
O1—Eu1—N4	129.29 (9)	C12—C13—C8	109.6 (3)
O3—Eu1—N4	146.89 (9)	N2—C13—H13	108.3
O7—Eu1—N4	77.08 (9)	C12—C13—H13	108.3
O8—Eu1—N4	24.95 (9)	C8—C13—H13	108.3
O13—Eu1—N4	71.13 (10)	N2—C14—C15	122.7 (3)
O5—Eu1—N4	127.65 (9)	N2—C14—H14	118.6
O10—Eu1—N4	24.77 (9)	C15—C14—H14	118.6
O4—Eu1—N4	97.84 (8)	C16—C15—C20	119.4 (3)
O11—Eu1—N4	85.35 (11)	C16—C15—C14	119.8 (3)
O2—Eu1—N4	85.53 (8)	C20—C15—C14	120.7 (3)
N3—Eu1—N4	102.20 (10)	C17—C16—C15	120.6 (3)
C6—O1—Eu1	132.23 (19)	C17—C16—H16	119.7
C1—O2—C22	116.9 (3)	C15—C16—H16	119.7
C1—O2—Eu1	115.61 (18)	C16—C17—C18	120.9 (3)
C22—O2—Eu1	127.2 (2)	C16—C17—H17	119.6
C20—O3—Eu1	125.56 (19)	C18—C17—H17	119.6
C19—O4—C21	116.2 (3)	C19—C18—C17	119.9 (3)
C19—O4—Eu1	116.49 (18)	C19—C18—H18	120.1
C21—O4—Eu1	126.9 (2)	C17—C18—H18	120.1
N3—O5—Eu1	95.9 (2)	C18—C19—O4	126.0 (3)
N3—O7—Eu1	97.2 (2)	C18—C19—C20	120.9 (3)
N4—O8—Eu1	98.1 (2)	O4—C19—C20	113.1 (3)
N4—O10—Eu1	96.2 (2)	O3—C20—C15	122.1 (3)
N5—O11—Eu1	95.5 (2)	O3—C20—C19	119.5 (3)
N5—O13—Eu1	100.0 (2)	C15—C20—C19	118.4 (3)
C7—N1—C8	126.2 (3)	C15—C20—Eu1	156.5 (2)
C7—N1—H1N	116.9	C19—C20—Eu1	84.60 (19)
C8—N1—H1N	116.9	O4—C21—H21A	109.5
C14—N2—C13	128.7 (3)	O4—C21—H21B	109.5
C14—N2—H2N	115.6	H21A—C21—H21B	109.5
C13—N2—H2N	115.6	O4—C21—H21C	109.5
O6—N3—O7	122.0 (4)	H21A—C21—H21C	109.5
O6—N3—O5	121.9 (4)	H21B—C21—H21C	109.5
O7—N3—O5	116.1 (3)	O2—C22—H22A	109.5
O6—N3—Eu1	179.0 (4)	O2—C22—H22B	109.5
O7—N3—Eu1	57.55 (19)	H22A—C22—H22B	109.5
O5—N3—Eu1	58.57 (19)	O2—C22—H22C	109.5
O9—N4—O10	121.2 (4)	H22A—C22—H22C	109.5
O9—N4—O8	123.0 (4)	H22B—C22—H22C	109.5
O10—N4—O8	115.8 (3)	C2M—O1M—H1O	107.9
O9—N4—Eu1	177.3 (3)	O1M—C2M—H2MA	108.5
O10—N4—Eu1	58.99 (17)	O1M—C2M—H2MB	108.6
O8—N4—Eu1	56.91 (17)	H2MA—C2M—H2MB	107.6
O12—N5—O11	123.7 (4)	O1M—C2M—H2MC	114.8
O12—N5—O13	120.6 (4)	H2MA—C2M—H2MC	108.6
O11—N5—O13	115.8 (4)	H2MB—C2M—H2MC	108.6
O12—N5—Eu1	176.0 (3)		

### Hydrogen-bond geometry (Å, °)

**Table:**

*D*—H···*A*	*D*—H	H···*A*	*D*···*A*	*D*—H···*A*
N1—H1N···O1	0.86	1.88	2.575 (3)	137
N2—H2N···O3	0.86	1.88	2.593 (3)	139
O1M—H1O···O13	0.85	2.18	2.993 (6)	160
